# Balance impairment in patients with moderate-to-severe traumatic brain injury: Which measures are appropriate for assessment?

**DOI:** 10.3389/fneur.2022.906697

**Published:** 2022-08-03

**Authors:** Julie M. Joyce, Chantel T. Debert, Mathilde Chevignard, Gilad Sorek, Michal Katz-Leurer, Isabelle Gagnon, Kathryn J. Schneider

**Affiliations:** ^1^Department of Radiology, Cumming School of Medicine, University of Calgary, Calgary, AB, Canada; ^2^Alberta Children's Hospital Research Institute, University of Calgary, Calgary, AB, Canada; ^3^Hotchkiss Brain Institute, University of Calgary, Calgary, AB, Canada; ^4^Integrated Concussion Research Program, University of Calgary, Calgary, AB, Canada; ^5^Department of Clinical Neurosciences, Cumming School of Medicine, University of Calgary, Calgary, AB, Canada; ^6^Laboratoire d'Imagerie Biomédicale, LIB, Inserm, CNRS, Sorbonne Université, Paris, France; ^7^GRC 24 HaMCRe, Handicap Moteur et Cognitif & Réadaptation, Sorbonne Université, Paris, France; ^8^Rehabilitation Department for Children with Acquired Neurological Injury, Saint Maurice Hospitals, Saint Maurice, France; ^9^Department of Physical Therapy, Sackler Faculty of Medicine, Tel Aviv University, Tel Aviv, Israel; ^10^Montreal Children's Hospital Trauma Center, McGill University Health Center, Montreal, QC, Canada; ^11^Faculty of Medicine and Health Sciences, School of Physical and Occupational Therapy, McGill University, Montreal, QC, Canada; ^12^Division of Pediatric Emergency Medicine, Faculty of Medicine and Health Sciences, McGill University, Montreal, QC, Canada; ^13^Sport Injury Prevention Research Centre, Faculty of Kinesiology, University of Calgary, Calgary, AB, Canada; ^14^Evidence Sport and Spinal Therapy, Calgary, AB, Canada

**Keywords:** traumatic brain injury, balance, gait, postural stability, dual-task

## Abstract

Left untreated, balance impairment following moderate-to-severe traumatic brain injury (TBI) can be highly debilitating and hinder activities of daily life. To detect impairments, clinicians need appropriate assessment tools. The objective of this study was to evaluate the feasibility and utility of a battery of clinical balance assessments in adults with moderate-to-severe TBI within 6-months of injury. Thirty-seven adults with TBI [Glasgow Coma Scale score ≤ 12 (33 M/4 F) age 18–50 years] participated in balance testing. Assessments included the Balance Error Scoring System (BESS), National Institutes of Health Standing Balance Test (NIH-SBT), Functional Gait Assessment (FGA), Advanced Functional Gait Assessment (FGA-A), Tandem Gait Test (TGT), Berg Balance Scale (BBS), and Walking While Talking Test (WWTT). We identified pronounced ceiling effects on the BBS and FGA, two widely used clinical balance assessments. The NIH-SBT, WWTT, and FGA used in conjunction with the FGA-A, offered versatility in their capacity to assess patients across the balance severity spectrum. This study provides evidence to support a stepwise approach to balance assessment that can be adapted to the broad range of balance ability found in moderate-to-severe TBI.

## Introduction

Traumatic brain injury (TBI) is a growing public health concern affecting an estimated 69 million individuals globally each year (1). While the majority of TBIs are mild, moderate-to-severe TBI accounts for 19% of injuries sustained (1, 2). Recent work investigating long-term outcomes 8-years following severe TBI has found a high prevalence of patients reporting balance impairment (3, 4). Yet, it has also been shown that individuals with moderate-to-severe TBI tend to underestimate balance deficits (5). Balance is integral to daily function and TBI patients with poor balance are at high risk for re-injury. Earlier commencement and greater intensity of neurorehabilitation following moderate-to-severe TBI is associated with better functional outcomes (6–9). As such, identifying and addressing balance impairments shortly after injury can help mitigate long-term functional impact.

There is limited research on specific tools for balance assessment in moderate-to-severe TBI. Often balance assessments are developed for a specific clinical population and their use is expanded to other clinical populations without thorough evaluation prior to their adoption. However, the utility of a particular assessment in one clinical population does not necessarily extend to a different population. Passing over the important evaluative step can lead to the use of inappropriate or insensitive assessment tools, ultimately leading to impairments going undetected. For treatment to be delivered to those who can benefit from balance interventions, clinicians need appropriate tools to identify deficits.

Underlying causes of balance impairment can be multifactorial. Neural control of balance is established through multiple interacting sensory, motor and cognitive systems (10). Visual, vestibular and proprioceptive systems are of high importance to balance function (11). Dysfunction of each has been linked to balance impairment (12–14) and all are susceptible to damage in TBI (15–17). Balance deficits can also manifest as a result of a failure to integrate information acquired by each of these systems. Accordingly, structures such as the cerebellum, a site of integration of proprioceptive, vestibular and visual input, are also of critical importance for balance (18). Comprehensive neurological exams can be performed to ascertain whether visual, vestibular, proprioceptive and cerebellar deficits are present. Determining the impact of different neurological impairments on balance performance in TBI patients may help to better understand factors contributing to balance impairment post-TBI and identify which patients need comprehensive balance testing.

The purpose of this study was to evaluate the feasibility and utility of a battery of clinical balance assessments in adults with moderate-to-severe TBI. The balance tests selected were widely used in mild TBI and/or clinical neurological populations but lacked extensive evaluation specifically in moderate-to-severe TBI. Secondly, we assessed the impact of neurological impairments and time since injury on balance scores to inform (1) which patients would benefit from balance interventions and (2) whether the utility of clinical assessments varied at different phases post-injury.

## Materials and methods

### Study design

This research was part of the multi-site international SiMPly Rehab initiative that aimed to identify alterations in oculomotor, vestibulo-ocular and balance function that occur following TBI of all severities (mild, moderate and severe) in children, youth and adults. Data presented in this sub-study was a cross-sectional analysis focused on assessment of balance in the subset of adults with moderate-to-severe TBI. The study protocol was registered at clinicaltrials.gov (NCT03215082) and was approved by the University of Calgary Conjoint Health Research Ethics Board, McGill University Health Center Research Ethics Board, Comité de Protection des Personnes Île-de-France 1 and the Helsinki ethics committees of the Alyn Children and Adolescent Rehabilitation Hospital, Loewenstein Rehabilitation Center and Tel Aviv University. Written informed consent was obtained for all subjects. When subjects were deemed incompetent due to cognitive impairment subsequent to their TBI, a surrogate provided written informed consent on their behalf.

### Participants

Criteria for inclusion was adults aged 18 to 50 years of age who were diagnosed with a moderate or severe TBI and were within 6 months of injury. TBI severity was defined using the Glasgow Coma scale (GCS) with a lowest score within the first 24 h of injury ≤ 12 classified as moderate-to-severe TBI (19). Individuals were approached to participate as soon as they were cleared by a physician to actively participate in rehabilitation. Participants were excluded if they sustained another TBI in the preceding 6 months, or any previous TBI with unresolved symptoms or impairments. Participants from hospitals and neurorehabilitation centers in Canada (Foothills Medical Centre), France (5 rehabilitation centers within the Île-de-France region) and Israel (Alyn Children and Adolescent Rehabilitation Hospital and Loewenstein Rehabilitation Center) were evaluated between the years 2018–2020. As one of the aims of the broader SiMPly Rehab initiative was to evaluate feasibility of different oculomotor, vestibulo-ocular and balance assessments in the moderate-to-severe TBI population, participants were recruited even if co-existing injuries and impairments prevented participation in certain components (e.g., balance assessment battery) of the SiMPly Rehab study.

### Neurological exam

All participants underwent an in-depth neurological exam performed by a physician specialized in physical medicine and rehabilitation or a physiotherapist. The neurological exam included:

#### Cranial nerve assessment

- CN I–Identification of odor (eyes closed)
- CN II–Tested visual fields and visual acuity
- CN III, IV, VI–Tested pupillary reflex and convergence; assessed smooth pursuits and saccades for asymmetry, lack of coordinated movement and nystagmus
- CN V–Assessed facial sensation in regions innervated by each of the three divisions of the trigeminal nerve through light touch; tested ability to clench jaw
- CN VII–Symmetric movement of facial muscles, identification of taste with anterior tongue
- CN VIII–Head thrust test, whisper test with occlusion of opposite ear
- CN IX and CN X–Ability to swallow and say “ah” with tongue depressor, gag reflex
- CN XI–Ability to resist head rotation and to elevate shoulders against resistance
- CN XII–Capacity to stick out tongue, move tongue rapidly side to side

#### Cerebellar scan

- Upper extremity dysdiadochokinesia–Ability to perform rapid alternating movement of hands on thighs for 10 s
- Lower extremity dydiadochokinesia–Ability to perform rapid tapping of feet on floor for 10 s
- Upper extremity dysmetria–Finger-to-nose test (minimum of five repetitions in 5 s)
- Lower extremity dysmetria–Heel-to-shin test (minimum of five smooth repetitions on each side in rapid succession)

#### Long tract signs

- Upper extremity clonus–Examiner supported elbow in a partly flexed position, wrist was rapidly extended and observed for rhythmic clonic movements
- Lower extremity clonus–Examiner supported knee in a partly flexed position, foot was dorsiflexed and observed for rhythmic clonic movements
- Babinski reflex–Sole of each foot was stroked using a blunt instrument and toe response was observed

#### Reflex examination

- Biceps, triceps and Achilles tendon–Reflexes tested with hammer; absence of a reflex, hyperreflexia and clonic movements noted

#### Dermatomes assessment

- Upper and lower extremity dermatomes–Tested for abnormal or asymmetric sensation in response to light touch

#### Key muscles examination

- Restricted isometric movement testing–Used to assess muscle groups including hip flexors, knee extensors, dorsiflexors, shoulder abductors, elbow flexors, elbow extensors, wrist extensors and interosseous muscles to detect abnormal muscle weakness and/or asymmetry

Additionally, the presence of proprioceptive deficits (Romberg test) visuospatial neglect and any general movement disorders such as ataxia, hemiparesis, quadriparesis, spasticity and hypotonia were noted. Readers are directed to Bickley et al. (20) for further detail on the neurological examination (20). Participants' overall level of disability was assessed using the Glasgow Outcome Scale Extended (GOS-E) (21). The GOS-E is a structured interview that considers the functional consequences of TBI and is used to classify global outcomes following injury.

### Balance assessment battery

The SiMPly Rehab balance assessment battery included the Balance Error Scoring System (BESS) (22), National Institutes of Health Standing Balance Test (NIH-SBT) (23), Functional Gait Assessment (FGA) (24), Advanced Functional Gait Assessment (FGA-A) (25), Tandem Gait Test (TGT) (26), Berg Balance Scale (BBS) (27), and Walking While Talking Test (WWTT) (28). Protocols and scoring for each test are described in Table 1. Participants were excluded from specific tests if they had co-existing injuries or impairments (e.g., cervical fracture, orthopedic injury, severe cognitive impairment that impacted comprehension of task instructions, etc.) that limited their ability to complete the assessment safely. Due to lack of equipment, the NIH-SBT was not completed in Israel. Testing typically began with assessments of postural stability (BESS, NIH-SBT) followed by dynamic balance testing (TGT, FGA, FGA-A) and dual-task tests (WWTT) depending on which assessments participants could feasibly complete. Often testing was completed in a single session but in certain cases where participants exhibited or expressed fatigue, testing was carried out over multiple visits. All tests were completed within a week.

**Table 1:** Descriptive summary of balance test protocols and scoring of assessments included in the SiMPly Rehab balance testing battery.

**Assessment**	**Protocol**	**Scoring**
BESS (22, 29, 30)	**6 poses (eyes closed, barefoot, 20 s each, hands on hips)** 1) double leg stance (feet together), hard surface 2) single leg stance (stand on non-dominant foot), hard surface 3) tandem leg stance (non-dominant foot in back), hard surface 4) double leg stance (feet together), foam surface 5) single leg stance (stand on non-dominant foot), foam surface 6) tandem leg stance (non-dominant foot in back), foam surface	Participants are scored on the number of errors made (deviations from proper stance) for a maximum of 10 errors per pose. Errors included: Lifting hands off iliac crests, opening eyes, stepping, stumbling or falling, remaining out of the proper test position for more than 5 s, moving hip into more than 30° of flexion or abduction, lifting forefoot or heel. For full scoring details see Riemann et al. (22), *JSR*.
NIH-SBT (23, 31)	**5 poses (50 s each, arms crossed on chest)** 1) feet together, eyes open, hard surface 2) feet together, eyes closed, hard surface 3) feet together, eyes open, foam surface 4) feet together, eyes closed, form surface 5) tandem stance, eyes open, hard surface *Stop rules are incorporated into the application*.	Scores are generated automatically by NIH software. Three scores were used for data analysis: 1. age-corrected standard score (normative mean = 100, SD = 15) 2. ratio 1 = pose 2 / pose 1 3. ratio 2 = pose 4 / pose 1 Lower age-corrected scores are indicative of greater motor dysfunction. Lower ratios reflect better performance.
FGA (24, 32)	**10 tasks** 1) gait on level surface 2) gait with changes in walking speed 3) gait with horizontal head turns 4) gait with vertical head turns 5) gait with pivot turn 6) step over obstacle 7) gait with narrow base of support 8) gait with eyes closed 9) gait while ambulating backwards 10) steps	Performance for each task is scored on an ordinal scale from 0 to 3 with lower scores indicative of greater impairment.
FGA-A (25)	**6 tasks** 1) tandem gait with head motion 2) tandem gait backwards 3) walking with rapid horizontal head turns 4) walking with rapid vertical head turns 5) walking backwards with rapid horizontal head turns 6) walking backwards with rapid vertical head turns	Performance for each task is scored on an ordinal scale from 0 to 3 with lower scores indicative of greater impairment.
TGT (26)	**4 trials** Walk heel-to-toe for 3 m and return to starting point (time recorded), repeat 3 times (four trials total)	Best time of four trials used for scoring. Shorter times reflect better performance.
BBS (33)	**14 tasks** 1) sitting to standing 2) standing unsupported 3) sitting with back unsupported with feet supported on floor or stool 4) standing to sitting 5) transfers (between chairs) 6) standing unsupported with eyes closed 7) standing unsupported with feet together 8) reaching forward with outstretched arm while standing 9) pick up object from the floor in a standing position 10) turning to look behind over left and right shoulders while standing 11) turn 360 degrees	Performance of each task is scored on a five-point scale range from 0 to 4. A score of 0 is indicative of the lowest level of function and a score of 4 is indicative of the highest level of function.
	12) place alternate foot on step or stool while standing unsupported 13) standing unsupported on foot in front 14) standing on one leg	
WWTT (28, 34)	**3 tasks** 1) WWTT-gait: Walk 6 m at normal pace and return to starting point. 2) WWTT-simple: Walk 6 m at normal pace and return to starting point while reciting the alphabet. 2) WWTT-complex: Walk 6 m at normal pace and return to starting point while reciting alternating letters of the alphabet. *Time recorded for all tasks*.	Reduced performance in dual task conditions (task 2 and 3) is described as dual task cost (DTC). Relative DTC is calculated as follows: $DTC\;=\;\frac{Dual\;task\;time-WWTTgait\;time}{WWTTgait\;time}\;x\;100$

Detailed descriptions of protocols and scoring can be found in references cited for each assessment. BESS, Balance Error Scoring System; NIH-SBT, National Institutes of Health Standing Balance Test; FGA, Functional Gait Assessment; FGA-A, Advanced Functional Gait Assessment; BBS, Berg Balance Scale; TGT, Tandem Gait Test; WWTT, Walking While Talking Test.

### Statistical analysis

Descriptive statistics (median, interquartile range (IQR) and proportion as appropriate) were used to summarize patient characteristics (age, sex, time since injury, GCS, coma duration, GOS-E, number of previous TBI, imaging findings), neurological exam results and scores achieved on the balance assessment battery. To evaluate feasibility of assessments, the percentage of participants that completed each balance assessment was calculated. Balance score distributions were evaluated for significant departures from normality (Shapiro-Wilk test) as well as floor and ceiling effects. Associations between scores of different balance assessments were evaluated using Spearman's correlation. Following common practice, correlation strength was operationally defined as |r_s_| < 0.4 = weak, |r_s_| = 0.4–0.7 = moderate and |r_s_| > 0.7 = strong (35, 36).

The influence of specific neurological deficits pertinent to balance function (including cranial nerve III, IV or VI palsies, cranial nerve VIII palsy, cerebellar and proprioceptive deficits) on balance was examined by (a) plotting balance score distributions of patients with specific neurological impairments over balance score distributions of the full sample and (b) calculating median and IQR/range of each subset.

To examine the impact of time since injury, participants were divided into three groups (i) 2-weeks to 1 month, (ii) >1 month to 3 months and (iii) >3 months to 6 months post-injury and balance scores (median, IQR/range) for each group were calculated. Non-parametric Mann-Whitney U tests were used to compare participants assessed at different time periods post-injury in groups where n > 5. All statistical analyses were performed using IBM SPSS 28 (Armonk, New York).

## Results

### Patient characteristics

Forty-four adults with moderate-to-severe TBI (<6 months post-injury) were recruited for the SiMPly Rehab initiative. Six participants were ineligible for balance assessment due to co-existing injuries and impairments (3 required mobility aids (2 wheelchair, 1 cane), 1 femur fracture, 1 apraxia, 1 severe cognitive impairment). Additionally, one participant was unwilling to complete balance testing due to high TBI symptom burden.

Thirty-seven adults with moderate-to-severe TBI (33 males, 4 females) of median age 26.52 years (IQR 20.77–35.70) 2 weeks-6 months post-injury participated in the balance component of the SiMPly Rehab study. Patient characteristics are further described in Table 2. Participants exhibited a wide variety of neurological impairments including palsies of cranial nerve III, IV, VI and VIII (integral for oculomotor and vestibular function), evidence of cerebellar impairment (dysmetria, dysdiadochokinesia, ataxia), proprioceptive deficits and spasticity. Neurological exam results can be found in Table 3.

**Table 2:** Patient characteristics of adults with TBI who participated in balance testing (*n* = 37).

	**Median**	**IQR**	**Min**	**Max**
Sex, M/F	33 M/4 F	-	-	-
Age (years)	26.52	20.77–35.70	18.36	50.83
Time since injury (days)	93	68–118	18	182
GCS (post-injury)	5	3–7	3	12
GOS-E (at assessment)	5	4–5	3	8
Coma duration (days)	7	2–15	0	35
Current living situation, *n* (%)				
Home	13 (35.1)			
Rehabilitation hospital	24 (64.9)			
Site of assessment, *n* (%)				
Canada	9 (24.3)			
France	25 (67.6)			
Israel	3 (8.1)			
Evidence of skull fracture, *n* (%)				
Yes	22 (50.0)			
No	7 (15.9)			
Not reported	15 (34.1)			
Evidence of intracranial injury, *n* (%)				
Epidural hematoma	2 (5.4)			
Extraaxial hematoma	1 (2.7)			
Subdural hematoma	6 (16.2)			
Subarachnoid hemorrhage	5 (13.5)			
Contusion	2 (5.4)			
Intracerebral hemorrhage	16 (43.2)			
Diffuse axonal injury	4 (10.8)			
Not reported	1 (2.7)			

GCS, Glasgow Coma Scale; GOS-E, Glasgow Outcome Scale Extended.

**Table 3:** Neurological exam results of individuals who participated in balance testing.

**Assessment**	**Tested, *n***	**Abnormal, *n* (%)**
**Cranial nerve exam**		
CN I	37	0 (0.0)
CN II	37	2 (5.4)
CN III	37	6 (16.2)
CN IV	37	5 (13.5)
CN V	35	5 (14.2)
CN VI	37	2 (5.4)
CN VII	37	7 (18.9)
CN VIII	37	3 (8.1)
CN IX	37	2 (5.4)
CN X	37	1 (2.7)
CN XI	37	2 (5.4)
CN XII	36	0 (0.0)
**Cerebellar signs**		
Dysdiadochokinesia UE	36	8 (22.0)
Dysdiadochokinesia LE	35	6 (17.1)
Dysmetria UE	36	7 (19.4)
Dysmetria LE	35	6 (17.1)
Ataxia	37	3 (8.1)
**Long tract signs**		
Clonus UE	36	1 (2.8)
Clonus LE	36	9 (2.5)
Babinski	35	5 (14.3)
**Proprioception**	36	4 (11.1)
**Reflexes**		
Biceps	37	12 (32.4)
Triceps	37	11 (29.7)
Achilles	37	10 (27.0)
**Key muscles**		
Hip flexors	35	6 (17.1)
Knee extensors	35	8 (22.9)
Dorsiflexors Shoulder abductors	35 35	8 (22.9) 10 (28.6)
Elbow flexors	35	9 (25.7)
Elbow extensors	35	11 (26.8)
Wrist extensors	35	10 (28.6)
Interosseous muscles	35	11 (31.4)
**Dermatomes**		
Light touch UE	37	6 (16.2)
Light touch LE	37	3 (8.1)
**General movement disorders**		
Hemiparesis	37	6 (16.2)
Quadriparesis	37	2 (5.4)
Spasticity	35	9 (25.7)
Hypotonia	35	1 (2.9)
Visuospatial neglect	37	1 (2.7)
Hemianopia	32	5 (15.6)
Visual extinction	31	0 (0.0)

CN, cranial nerve; LE, lower extremity; UE, upper extremity.

### Evaluation of balance assessment battery

#### Feasibility

A large majority of participants were able to complete the FGA (94.6%), BBS (94.6%) and the first two poses of the NIH-SBT (100%). Generally, participants were more challenged by the BESS, FGA-A, TGT, WWTT and final three poses of the NIH-SBT which could only be completed by 67.6–79.4% of the cohort. While all assessments were attempted, tests were not completed if they could not be performed safely due to severe balance impairment. Number of participants able to complete each balance assessment is reported in Table 4.

**Table 4:** Descriptive summary of clinical balance assessment feasibility and test scores.

**Assessment**	**Attempted, *n***	**Completed, *n* (%)**	**Scoring range**	**Median score**	**IQR**	**Shapiro-Wilk, W (p)**	**Impairment threshold**	**Impaired, *n* (%)**
BESS	37	28 (75.7)	0–60	19	11–34	0.949 (0.192)	<10^th^ percentile (healthy adults) *age-dependent* <2^nd^ percentile (healthy adults) *age-dependent*	15 (53.6) 9 (32.1)
NIH-SBT_age−corrected_	34	34 (100.0)	59–140	83	72–90	0.949 (0.112)	<10^th^ percentile ( ≤ 80-healthy adults) ≤ 2^nd^ percentile ( ≤ 70-healthy adults)	15 (42.8) 5 (14.3)
NIH-SBT${}_{\text{ratio}1}^{\text{a}}$	34	34 (100.0)	-	1.35	1.16–1.51	0.989 (0.979)	Undefined	-
NIH-SBT_ratio2_	34	27 (79.4)	-	2.57	1.94–3.50	0.925 (0.052)	Undefined	-
FGA	37	35 (94.6)	0–30	28	18–29	0.801 (<0.001)	≤ 27 (subnormal function–healthy adults) ≤ 22 (fall risk–older adults)	15 (42.8) 11 (31.4)
FGA-A	37	25 (67.6)	0–18	12	9–17	0.898 (0.017)	Undefined	-
TGT^a^	37	25 (67.6)	-	28 s	24–40 s	0.880 (0.008)	> 14 s (athletes with mild TBI)	25 (100.0)
BBS	37	35 (94.6)	0–56	56	53–56	0.654 (<0.001)	≤ 45 (fall risk–older adults)	4 (10.8)
WWTT${}_{\text{simple}}^{\text{b}}$	37	29 (78.4)						
Time			-	14 s	10–17 s	0.792 (<0.001)	≥ 20 s (fall risk–older adults)	4 (13.8)
Dual task cost			-	0%	0–17%	0.859 (0.002)	Undefined	-
WWTT${}_{\text{complex}}^{\text{b}}$	37	29 (78.4)						
Time			-	18 s	13–24 s	0.810 (<0.001)	≥ 33 s (fall risk–older adults)	4 (13.8)
Dual task cost			-	29%	14–56%	0.919 (0.038)	Undefined	-

Existing thresholds of impairment and target population are described. Balance Error Scoring System (BESS) thresholds vary with age and can be found in Iverson et al. (29). Scoring references are included in Table 1. ^a^1 extreme outlier (> 3^*^IQR from Q1 or Q3) excluded from analyses. ^b^2 extreme outliers excluded from analyses. Q1, first quartile; Q3, third quartile.

#### Score distributions

**Figure 1:**
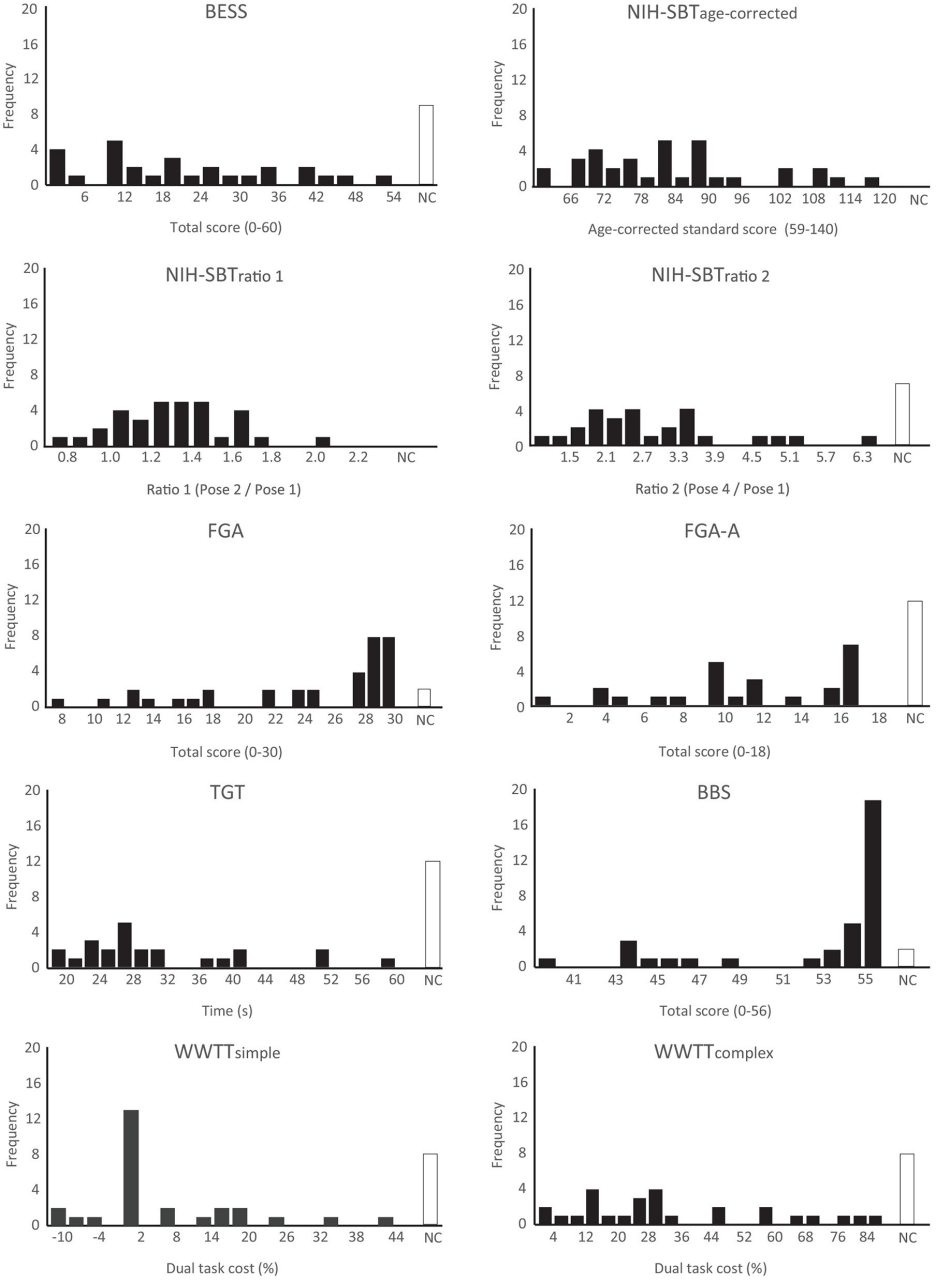
Histograms presenting score distributions of all balance measures included in the SiMPly Rehab testing battery. One extreme outlier (> 3*IQR from Q1 or Q3) was excluded on the NIH-SBT_ratio1_ and TGT. Two extreme outliers were excluded on the WWTT-simple and WWTT-complex. NC: Assessment not completed; Q1: first quartile; Q3: third quartile.

Significant departures from normality were detected on the BBS, TGT, FGA, FGA-A, WWTT-simple and WWTT-complex. Ceiling effects were evident on the FGA and BBS where 16/35 (45.7%) and 24/35 (68.6%) of participants who completed the assessments achieved the maximum or second highest possible score. Departures from normality, floor and ceiling effects were not found on the BESS or NIH-SBT. Median scores achieved on all balance assessments as well as Shapiro-Wilk test results are reported in Table 4. Balance score distributions can be seen in Figure 1.

#### Associations across balance measures

Spearman's correlations between balance tests included in the assessment battery ranged from −0.585 to 0.739. The strongest significant correlations were between the FGA and the FGA-A (r_s_ = 0.733) and the FGA and BBS (r_s_ = 0.739). Twelve other significant correlations of moderate strength (0.4 ≤ |r_s_| ≤ 0.7) were identified between balance measures. Specifically, the BESS was correlated with the NIH-SBT_age−corrected_ (r_s_ = −0.417), NIH-SBT_ratio1_ (r_s_ = 0.506), FGA (r_s_ = −0.546) and FGA-A (r_s_ = −0.550). The NIH-SBT_age−corrected_ was correlated with the FGA (r_s_ = 0.465), TGT (r_s_ = −0.527), WWTT_simple_ (r_s_ = −0.448) and BBS (r_s_ = 0.640). The FGA was correlated with the TGT (r_s_ = −0.576). The FGA-A was correlated with the TGT (r_s_ = −0.473) and WWTT_complex_ (r_s_ = −0.463). Lastly, the TGT was correlated with the BBS (r_s_ = −0.585). All correlational analyses are reported in Table 5.

**Table 5:** Spearman correlation matrix across balance assessments.

	**BESS**	**NIH-SBT _age−corrected_**	**NIH-SBT _ratio1_**	**NIH-SBT _ratio2_**	**FGA**	**FGA-A**	**TGT**	**BBS**	**WWTT _simple_**	**WWTT _complex_**
BESS	-									
NIH-SBT_age−corrected_	−0.417*	-								
NIH-SBT${}_{\text{ratio}1}^{\text{a}}$	0.506**	0.138	-							
NIH-SBT_ratio2_	−0.046	0.248	0.344	-						
FGA	−0.546**	0.465**	−0.149	−0.022	-					
FGA-A	−0.550**	0.390	−0.220	−0.073	0.733***	-				
TGT^a^	0.068	−0.527**	−0.245	−0.195	−0.576**	−0.473*	-			
BBS	−0.282	0.640***	0.001	−0.040	0.739***	0.325	−0.585**	-		
WWTT${}_{\text{simple}}^{\text{b}}$	−0.001	−0.448*	−0.088	−0.273	−0.316	−0.213	0.282	−0.362	-	
WWTT${}_{\text{complex}}^{\text{b}}$	0.071	−0.140	−0.048	−0.317	−0.395*	−0.463*	0.390	−0.101	0.313	-

All analyses were two-tailed. Relative dual task cost was used for WWTT-simple and WWTT-complex correlational analyses. Dashes along the diagonal represent perfect correlation (r_s_ = 1). ^*^ p < 0.05; ^**^ p < 0.01; ^***^ p < 0.001. ^a^1 extreme outlier (> 3^*^IQR from Q1 or Q3) excluded from analyses. ^b^2 extreme outliers excluded from analyses. Q1, first quartile; Q3, third quartile.

### Potential moderators of balance performance

#### Neurological impairment

Of the participants presenting with cerebellar signs (*n* = 12), only 4/12 were able to safely complete the TGT. While a few additional participants (7/12) could complete the BESS, the number of errors made was high (median = 42, IQR = 18–48) relative to the full sample (median = 19, IQR = 11–34). Additionally, 11/12 participants with cerebellar impairment completed the FGA of which 8/11 exhibited subnormal dynamic balance ability and 6/11 reached the threshold for fall risk (see Table 4 for scoring cut-offs).

Otherwise, broadly, balance score distributions of participants presenting with neurological impairments did not show evidence of deviation from the distribution of the full sample. Score distributions of participants exhibiting cranial nerve III, IV or VI palsies, cranial nerve VIII palsy, cerebellar impairment and proprioceptive deficits relative to the full sample can be seen in Supplementary Figures 1–4 and descriptive score summaries can be found in Supplementary Table 1.

#### Time since injury

Four adults with TBI participated in balance assessments within 1 month of injury. Median scores achieved by this subset of participants were consistent with the full sample apart from the FGA and NIH-SBT_ratio2_ (FGA median score = 19; NIH-SBT_ratio2_ = 4.16). Of the remaining participants, 15 completed assessments between 1- and 3-months post-injury (FGA median = 29; NIH-SBT_ratio2_ = 2.40) and 18 completed assessments between 3- and 6- months post-injury (FGA median = 29; NIH-SBT_ratio2_ = 2.82).

Mann-Whitney U tests revealed no significant differences across balance measures in participants assessed between 1- and 3-months compared to those assessed between 3- and 6-months post-injury. Balance score distributions of all groups can be seen in Supplementary Figure 5. Median scores achieved on all balance measures and number of participants able to complete each assessment are stratified by time since injury in Supplementary Table 2.

## Discussion

This study presents a descriptive evaluation of a battery of balance assessments in adults with moderate-to-severe TBI in the sub-acute phase post-injury. We identified pronounced ceiling effects on the BBS and FGA, two broadly used clinical balance assessments. More challenging assessments, including the BESS, FGA-A, TGT and WWTT could only be completed by a subset of participants. The NIH-SBT was found to be a versatile assessment tool as it was highly feasible and did not exhibit ceiling effects. This study provides evidence to support a stepwise approach for balance assessment in moderate-to-severe TBI.

One of the primary aims of this study was to evaluate the feasibility of different clinical balance assessments in moderate-to-severe TBI. Factors that prohibited balance testing included physical impairments that necessitated the continued use of mobility aids as well as apraxia and severe cognitive impairment that impacted participants' ability to follow task instructions. Of participants who were able to undergo balance testing, feasibility varied depending on the task.

The BBS and FGA were highly feasible but had limitations. Initially, the BBS was developed to evaluate balance ability in the elderly and patients with acute stroke (27). Use of the BBS has been expanded to include patients diagnosed with multiple sclerosis (37), spinal cord injury (38), Parkinson's disease (39) and TBI (40) and is often used in clinical practice to predict risk of falls. Newstead et al. found excellent test-retest reliability of the BBS in TBI (40). However, the authors noted that TBI patients achieved high scores and cautioned against potential ceiling effects.

The FGA was developed for patients with vestibular disorders as a modification of the Dynamic Gait Index (DGI) and was designed to reduce the ceiling effect seen on the DGI (24). Like the BBS, the FGA has since been used for other clinical populations including stroke (41–43) and mild TBI (44, 45). In this study, we found a ceiling effect on both the FGA and BBS which brings into question their utility in this population. For the FGA, this issue can be addressed through incorporation of the recently developed FGA-A (25) which adds several more challenging items to the standard FGA. The FGA-A has not yet been validated in moderate-to-severe TBI and our work suggests it warrants further evaluation as a complement to the FGA. As for the BBS, this assessment may help identify patients at high risk for falls but our data suggests it should not be used in isolation to assess balance impairment.

Static balance was assessed using the BESS and NIH-SBT. The BESS was originally developed for mild TBI and has been shown to correlate well with other measures of postural stability (22, 46). Here we found that the BESS was too challenging to be completed safely by 17.6% of the cohort. In particular, participants struggled with poses that were completed on a foam surface. The NIH-SBT was moderately correlated with the BESS, although the association did not reach our threshold for statistical significance. The NIH-SBT had several advantages for moderate-to severe TBI. One strength was that even partial completion of the test (pose 1 and pose 2) generated an age-corrected standard score. As such, it could be used for participants with more severe balance impairment who were unable to safely complete the more challenging NIH-SBT foam poses. The breadth of the scale allowed for scores achieved by highly impaired participants to be compared to participants with superior balance performance. Overall, the NIH-SBT was more feasible than the BESS in this cohort.

The TGT is commonly used to evaluate athletes following sports-related mild TBI (26, 47). The heel-to-toe step of the TGT is a highly coordinated movement and has been shown to be adversely affected following mild TBI (47, 48). The TGT requires participants to walk as quickly as possible heel-to-toe along a 3 m walkway. The competing priorities of the task (need for speed and coordination) proved to be a challenge in this cohort. It was not uncommon for participants to attend to one priority at the expense of the other. Consequently, some completed the task rapidly but exhibited deviations from proper gait while others were attentive of movement execution but failed to be mindful of the instruction to complete the task quickly. Managing multiple priorities simultaneously relies on executive function (49) which is commonly affected following moderate-to-severe TBI (50, 51) and likely contributed to poor performance on the TGT. Consistent with the decline in performance seen on the FGA-A relative to the FGA, these findings suggest that cognitively demanding tasks are more affected following moderate-to-severe TBI.

Reduced movement automaticity as a result of brain injury can lead to increased reliance on cognitive resources to process and monitor walking (52). The WWTT was designed on the premise that a common adaptation when encountering a challenging environment is to reduce gait speed (53). Recently, Rachal et al. found that the WWTT was clinically feasible and demonstrated excellent intra- and inter-rater reliability in inpatients with TBI who were receiving treatment at a rehabilitation hospital (54). However, the authors noted that the WWTT-simple was too easy for their cohort as participants completed the task without observable challenge or decrease in gait speed. These results are consistent with our study where participants exhibited a median 29% reduction in gait speed on the WWTT-complex task but generally did not exhibit substantial gait speed reductions on the WWTT-simple task. Although not all adults with TBI in our study completed the WWTT, we maintain that there is value to this assessment. It may be best suited for the assessment of subtle deficits. In patients that can safely undergo the WWTT, our recommendation is for testing to begin with the WWTT-simple task. The WWTT-complex task serves as a complement for individuals who do not exhibit gait speed reductions on the simpler task.

In terms of clinical utility for the balance assessments evaluated in this study, there is a high importance for establishing appropriate scoring thresholds. Inconsistent and unspecific scoring cut-offs described in the literature were a limitation across many of the measures included here. Proposed scoring cut-offs for the BBS vary considerably (<40–52/56) even within the same clinical populations (27, 55–59). Using the original cut-off proposed by Berg et al. (<45/56), 10.8% of participants tested in our study were identified as a fall risk (27). However, multiple systematic reviews have concluded that the BBS alone is not useful for predicting falls and that it should be used in conjunction with other tests and measures to ascertain fall risk (59–61). Suggested cut-offs scores for the FGA in the literature are also varied. FGA thresholds for fall risk range from ≤ 15/30 and ≤ 18/30 (Parkinson's) (55, 62) to ≤ 22/30 (community dwelling older adults) (24). Reference data for the FGA suggests that >27/30 on the FGA would be considered a normal score in adults up to 60 years of age (24, 32). Using this cut-off, 42.8% of participants tested in this study exhibited subnormal dynamic balance ability.

Prevalence of poor static balance performance (scores <10^th^ percentile of normative data) ranged from 42.8% (NIH-SBT) to 53.6% (BESS) (29). As several participants with poor balance were unable to complete the BESS, it appears that the threshold for impairment is substantially lower on the BESS than the NIH-SBT. The BESS was designed as a low-cost method to assess subtle impairments in postural stability in athletes with sports-related mild TBI. The BESS has been scrutinized due to poor inter-rater reliability (63) and subjective scoring criteria (64). While the total BESS score (sum of errors across all poses) is most commonly used clinically, inter-rater reliability varies depending on the pose (46, 63) and is higher in more experienced raters (65). Given the BESS's limited feasibility and low threshold for impairment found here, the NIH-SBT may be preferable for the assessment of static balance in moderate-to-severe TBI. The NIH-SBT provided additional value through automatically-generated ratio scores which were included to tease apart the influence of various systems integral to balance. The first ratio score (pose 2 / pose 1) is indicative of postural stability in the absence of visual input. The second ratio score (pose 4 / pose 1) reflects the relative reduction in postural stability when visual and somatosensory input are simultaneously disrupted.

A TGT cut-off of >14 s has been used in the assessment of sports-related mild TBI (26). However, multiple studies have found a high rate of false positives in healthy adults suggesting that the 14 second cut-off is unlikely to be clinically useful in a non-athlete TBI population (66, 67). No participants in our cohort completed the test in <18 s which supports a lack of clinical utility for this threshold in moderate-to-severe TBI.

The WWTT does not have established cut-offs for moderate-to-severe TBI. The WWTT cut-offs for fall risk in older adults are ≥ 20 s on the WWTT-simple and ≥ 33 s on the WWTT-complex (28). Gait speed exhibits marked reductions with advancing age (68). Baseline differences in gait speed are a confound when using the time elapsed on a single task or the absolute difference (dual task time–baseline gait speed) for scoring and compromise the validity of inter-individual comparisons (68). It is, therefore, preferable to compare WWTT performance based on the relative cost of a cognitive task on gait speed which can be calculated using the equation described in Table 1 (34).

Another factor to consider when evaluating the clinical utility of assessment tools is time since injury. Of the four participants tested within 1 month of injury, all were able to complete the FGA but achieved scores below the normative threshold. This suggests that the FGA may be more discriminative acutely post-injury; moreover, it may be a useful test for monitoring dynamic balance ability in early weeks after TBI. In contrast, two participants achieved near-maximal scores (53/56 and 54/56) on the BBS within the first month post-injury. Median balance scores and IQR of participants in the two groups assessed >1-month post-injury were largely similar suggesting that time since injury may not be driving variability in performance after the initial weeks following injury. This interpretation, however, is complicated by the many factors that can influence balance such as severity of injury (69, 70), age (71) and timing/intensity of rehabilitation (6, 7) which may mask temporal relationships with balance performance.

The strongest correlations between balance measures included the FGA and/or BBS. The ceiling effects of these assessments add uncertainty to the relationships we detected with other balance measures. While we expected performance to be related across measures, the assessments chosen were designed to evaluate different aspects of balance control (e.g., postural stability, dynamic balance, dual-task performance). As such, the twelve other correlations of moderate strength (0.4 ≤ |r_s_| ≤ 0.7) identified between other balance measures lend confidence to the construct validity of our battery.

A secondary aim of this study was to ascertain whether participants with specific neurological deficits would exhibit marked reductions in balance scores compared to the full sample. We found that participants with cerebellar impairment (dysmetria, dysdiadochokinesia and/or ataxia) exhibited poor performance on the FGA and BESS; additionally, a majority of this subset was unable to safely complete the TGT. As the cerebellum is integral to highly coordinated movement (72), it is perhaps unsurprising that tests involving complex gait sequences (FGA and TGT) were affected. Our results suggest that TBI patients presenting with cerebellar signs should undergo postural and dynamic balance testing, particularly given the elevated risk of falls seen in this subset of participants. Overall, we report that the BESS, FGA and TGT were sensitive to cerebellar impairment and the FGA was highly feasible even in this subset of participants.

With regards to other neurological deficits, we did not find evidence of balance score deviation from the distribution of the full sample. However, we had a limited sample of participants that exhibited certain deficits (e.g., only three participants presented with CN VIII palsy). Furthermore, impairment to individual systems (i.e., oculomotor dysfunction) may have been compensated for by other functional systems. For example, to an extent the influence of proprioceptive impairment on balance can be compensated for by vestibular function and vision (73). Participants with low scores may have had concurrent impairment of multiple systems responsible for balance. Future work with a larger sample can better address the question of which impairments (and/or combinations of impairments) are related to poor balance in TBI. In turn, this may help clinicians make judgements of which patients should undergo extensive balance testing following routine neurological exam.

### Proposed stepwise approach for balance testing

It is well-recognized that overall balance ability cannot be determined by evaluating a single aspect of balance control (74). As seen in this cohort, poor performance on one test does not necessarily imply poor performance on another. Thorough balance evaluations should include measures that target multiple domains of balance control (postural stability, dynamic balance, dual task ability, etc.) to understand an individual's unique combination of balance challenges. Our study highlights the various merits and limitations of different clinical balance assessments in a cohort of adults with moderate-to-severe TBI. A stepwise approach for multidimensional balance testing is offered below.

- Postural stability: The NIH-SBT has a predetermined sequence of poses that increase in difficulty throughout the test. Stop rules are incorporated into the application. If early poses are too challenging to complete, the testing can be terminated early in accordance with the stop rules. Scores are automatically generated even if individuals can only complete the easier poses.
- Dynamic balance: We recommend that testing begin with the FGA. If individuals achieve scores above the fall risk threshold (>22), the FGA-A can be introduced as an additional challenge with the evaluator monitoring closely. For those achieving scores in the “normal” range on the FGA (>27), inclusion of the FGA-A is strongly encouraged.
- Dual task: Testing should begin with the WWTT-simple and can proceed to the WWTT-complex which may reveal more subtle dual-task deficits.

Notably absent from these recommended measures are the BESS, TGT and BBS. As discussed previously, the BESS and TGT have traditionally been used for mild TBI and we found that these tests had limited feasibility in moderate-to-severe TBI. The TGT had additional concerns of validity given the participants' struggle with the competing priorities required by the task which was not captured in the scoring (measure of total time elapsed). Ceiling effects on the BBS, even within the first month post-injury, suggest this tool may not be clinically useful for moderate-to-severe TBI.

### Limitations

The SiMPly Rehab initiative was a multi-site study with multiple evaluators that carried out balance assessments. Although we did not assess inter-rater reliability, all evaluators were trained by experienced clinicians and followed a standardized manual of procedures. Participants recruited for the SiMPly Rehab initiative were a convenience sample and this may influence our estimates of balance impairment. Furthermore, for certain assessments, prevalence of impairment was determined using scoring cut-offs established for older adults and non-TBI clinical populations. Thus, the true incidence of balance impairment in TBI remains unclear. Our sample was largely male. As only 4 females were included in this cohort, we did not attempt to parse apart sex differences from other factors such as time since injury, neurological impairments, age, etc. Incidence of TBI is higher in males than females (75, 76). Thus, while a higher male:female ratio was expected, it may limit the generalizability of our findings. Additionally, in order not to disrupt recommended care, medication usage was not exclusionary. Several commonly used medications (including those for blood pressure, infections and seizures) can cause dizziness and light-headedness and may have contributed to poor balance performance (77). As detailed medication usage was not available for all participants, we did not examine the impact of medication usage on balance directly. Clinicians are urged to consider medication usage as a potential contributor to balance impairment as this may be a modifiable factor addressed by tailoring dosage or prescription.

### Considerations for future research

TBI is a heterogeneous injury and, as seen in this cohort, balance ability in individuals following TBI is highly variable. As such, assessments that can evaluate a broad spectrum of balance capability are needed. The NIH-SBT, FGA + FGA-A and WWTT offered versatility in their capacity to assess patients across the balance impairment severity spectrum. Our data provides preliminary insight into the value of different clinical balance assessments for adults with moderate-to-severe TBI. An important next step in evaluating the utility of these assessments will be to establish their reliability and validity in persons with moderate-to-severe TBI. The considerable variability in proposed impairment cut-off scores in the broader clinical literature suggests the need for population-specific thresholds. While not reviewed here, proposed minimal clinically important change for the measures included in our balance battery are also varied. Establishing meaningful change is essential to guide clinicians in monitoring of recovery (or lack thereof) and warrants further investigation. Another aspect of balance control, not explored in our study, are the reactive postural adjustments made to regain balance when stable posture is suddenly perturbed. Sections of the BESTest/Mini-BESTest/Brief-BESTest (5, 74, 78–80) evaluate this aspect of balance control and may provide additional value in assessing risk of falls in moderate-to-severe TBI.

## Conclusion

This study offers a multi-dimensional evaluation of balance in adults with moderate-to-severe TBI. We highlighted a broad spectrum of balance capability in this cohort and high incidence of deficits. These findings indicate a need for clinicians to precisely assess balance following TBI in order to implement targeted interventions. A selection of clinical assessments that probed different aspects of balance function were evaluated. We identified several tools (NIH-SBT, FGA + FGA-A, WWTT) that appear to be feasible and appropriate for the evaluation of balance in moderate-to-severe TBI using a stepwise approach. We provide evidence to support limited utility of the BBS, timed TGT and BESS in this population. Future work that establishes population-specific reliability, validity, meaningful scoring thresholds for impairment and minimal clinically important change is needed to help guide clinicians in the detection and monitoring of balance deficits post-TBI.

## Data availability statement

The datasets presented in this article are not readily available because we are restricted by our ethics agreement with the institutions participating in the SiMPly Rehab initiative as participants did not consent to this form of data sharing. Requests to access the datasets should be directed to KS, kjschnei@ucalgary.ca.

## Ethics statement

The studies involving human participants were reviewed and approved by the University of Calgary Conjoint Health Research Ethics Board, McGill University Health Center Research Ethics Board, Comité de Protection des Personnes Île-de-France 1 and the Helsinki Ethics Committees of the Alyn Children and Adolescent Rehabilitation Hospital, Loewenstein Rehabilitation Center and Tel Aviv University. Subjects provided their written informed consent to participate in this study. When subjects were deemed incompetent due to cognitive impairment subsequent to their TBI, a surrogate provided written informed consent on their behalf.

## Author contributions

JJ performed recruitment, data collection, analysis, writing the first draft of the manuscript, and continual editing of the manuscript. GS carried out recruitment, data collection, and editing of the manuscript. MK-L, MC, IG, and KS contributed to conception of the SiMPly Rehab initiative, organization, design, and execution as well as editing of the manuscript. CD contributed to data collection and, together with KS, was instrumental in the conceptualization, design, execution, and manuscript preparation for this sub-study. All authors contributed to the article and approved the submitted version.

## Funding

This study was funded by the European Research Area – the Network of European Funding for Neuroscience Research (ERA-NET NEURON) under grant number 13897 as part of the SiMPly Rehab initiative. JJ was funded by the Branch Out Neurological Foundation under grant numbers 10020638 and 10025578.

## Conflict of interest

The authors declare that the research was conducted in the absence of any commercial or financial relationships that could be construed as a potential conflict of interest.

## Publisher's note

All claims expressed in this article are solely those of the author and do not necessarily represent those of their affiliated organizations, or those of the publisher, the editors and the reviewers. Any product that may be evaluated in this article, or claim that may be made by its manufacturer, is not guaranteed or endorsed by the publisher.
