# Supplementary material for: Balance impairment in patients with moderate-to-severe traumatic brain injury: Which measures are appropriate for assessment?
Source: Front Neurol. 2022 Aug 3;13:906697. doi: 10.3389/fneur.2022.906697 (PMC9381921; doi:10.3389/fneur.2022.906697)
Supplement: Supplementary file 1 [file Data_Sheet_1.docx]

Supplemental Table 1. Descriptive summary of balance scores (median, interquartile range (IQR)) in subsets of participants with neurological deficits. Range (instead of IQR) was reported for subsets of participants where n < 5. Q1: first quartile; Q3: third quartile.

| Neurological sign | | | BESS | NIH-SBT  _age-corrected_ | NIH_ratio1_ | NIH_ratio2_ | FGA | FGA-A | TGT | BBS | WWTT  _simple_ | WWTT  _complex_ |
| --- | --- | --- | --- | --- | --- | --- | --- | --- | --- | --- | --- | --- |
| Full sample | N | Attempted | 37 | 34 | 34 | 34 | 37 | 37 | 37 | 37 | 37 | 37 |
|  |  | Completed | 28 | 34 | 34 | 27 | 35 | 25 | 25 | 35 | 29 | 29 |
|  | Median | | 19 | 83 | 1.35 | 2.57 | 28 | 12 | 28 s | 56 | 0% | 29% |
|  | IQR | Q1 | 11 | 72 | 1.16 | 1.94 | 18 | 9 | 24 s | 53 | 0% | 14% |
|  |  | Q3 | 34 | 90 | 1.51 | 3.50 | 29 | 17 | 40 s | 56 | 17% | 56% |
| CN III, IV and/or VI palsy  *see Suppl Fig 1* | N | Attempted | 10 | 10 | 10 | 10 | 10 | 10 | 10 | 10 | 10 | 10 |
|  |  | Completed | 7 | 10 | 10 | 6 | 10 | 6 | 8 | 9 | 9 | 9 |
|  | Median | | 23 | 81 | 1.39 | 2.82 | 25 | 10 | 34 s | 55 | 0% | 33% |
|  | IQR | Q1 | 12 | 72 | 1.24 | 1.86 | 17 | 7 | 27 s | 48 | 0% | 19% |
|  |  | Q3 | 33 | 88 | 1.46 | 4.23 | 28 | 13 | 41 s | 56 | 21% | 68% |
| CN VIII  palsy  *see Suppl Fig 2* | N | Attempted | 3 | 3 | 3 | 3 | 3 | 3 | 3 | 3 | 3 | 3 |
|  |  | Completed | 2 | 3 | 3 | 1 | 3 | 2 | 3 | 3 | 3 | 3 |
|  | Median | | 14 | 78 | 1.35 | 3.45 | 17 | 10 | 40 s | 46 | 25% | 56% |
|  | Range | Minimum | 2 | 60 | 1.07 | - | 16 | 10 | 29 s | 44 | 0% | 33% |
|  |  | Maximum | 26 | 87 | 1.50 | - | 24 | 10 | 60 s | 53 | 35% | 81% |
| Cerebellar  impairment  *see Suppl Fig 3* | N | Attempted | 12 | 10 | 10 | 10 | 12 | 12 | 12 | 12 | 12 | 12 |
|  |  | Completed | 7 | 10 | 10 | 6 | 11 | 7 | 4 | 10 | 8 | 8 |
|  | Median | | 42 | 78 | 1.47 | 2.23 | 22 | 8 | 29 s | 55 | 9% | 30% |
|  | IQR | Q1 | 18 | 67 | 1.26 | 1.63 | 13 | 4 | 27 s | 45 | 0% | 19% |
|  |  | Q3 | 48 | 92 | 1.67 | 2.92 | 28 | 16 | 38 s | 56 | 18% | 76% |
| Proprioceptive impairment  *see Suppl Fig 4* | N | Attempted | 4 | 4 | 4 | 4 | 4 | 4 | 4 | 4 | 4 | 4 |
|  |  | Completed | 3 | 4 | 4 | 2 | 4 | 2 | 3 | 4 | 4 | 4 |
|  | Median |  | 26 | 75 | 1.34 | 1.98 | 21 | 10 | 29 s | 50 | 0% | 52% |
|  | Range | Minimum | 12 | 60 | 1.30 | 1.70 | 17 | 10 | 23 s | 44 | -6% | 33% |
|  |  | Maximum | 34 | 88 | 1.50 | 2.26 | 29 | 10 | 40 s | 56 | 25% | 71% |

Supplemental Table 2. A descriptive summary of balance scores (median, IQR) at various times post-injury (score distributions can be visualized in Supplemental Table 5). Participants were divided into: i) 2 weeks-1-month post-injury (n = 4, dark blue); ii) >1 month to 3 months post-injury (n = 15, medium blue); and iii) >3 months to 6 months post-injury (n = 18, light blue). Range (instead of IQR) was reported for the participants within 1 month of injury due to small sample size (n < 5). * = one outlier (> 3*IQR from Q1 or Q3 of overall sample) excluded from analysis. IQR: interquartile range; Q1: first quartile; Q3: third quartile.

| Time since injury | | | BESS | NIH-SBT  _age-corrected_ | NIH_ratio1_ | NIH_ratio2_ | FGA | FGA-A | TGT | BBS | WWTT  _simple_ | WWTT  _complex_ |
| --- | --- | --- | --- | --- | --- | --- | --- | --- | --- | --- | --- | --- |
| 2 weeks - 1 month | N | Attempted | 4 | 4 | 4 | 4 | 4 | 4 | 4 | 4 | 4 | 4 |
|  |  | Completed | 2 | 4 | 4 | 2 | 4 | 2 | 4 | 3 | 4 | 4 |
|  | Median | | 14 | 81 | 1.22 | 4.16 | 19 | 11 | 29 s* | 53 | 8% | 29% |
|  | Range | Minimum | 2 | 73 | 1.07 | 3.45 | 14 | 10 | 28 s | 46 | -12% | 14% |
|  |  | Maximum | 26 | 87 | 1.35 | 4.87 | 24 | 12 | 60 s | 54 | 35% | 81% |
| >1 month - 3 months | N | Attempted | 15 | 14 | 14 | 14 | 15 | 15 | 15 | 15 | 15 | 15 |
|  |  | Completed | 12 | 14 | 14 | 12 | 14 | 11 | 10 | 14 | 13 | 13 |
|  | Median | | 20 | 86 | 1.31 | 2.40 | 29 | 10 | 29 s | 56 | 0%* | 29%* |
|  | IQR | Q1 | 5 | 71 | 1.20 | 1.89 | 18 | 7 | 22 s | 46 | 0% | 14% |
|  |  | Q3 | 33 | 91 | 1.59 | 3.00 | 29 | 17 | 34 s | 56 | 13% | 54% |
| >3 months - 6 months | N | Attempted | 18 | 16 | 16 | 16 | 18 | 18 | 18 | 18 | 18 | 18 |
|  |  | Completed | 14 | 16 | 16 | 13 | 17 | 12 | 11 | 18 | 12 | 12 |
|  | Median | | 19 | 83 | 1.42* | 2.82 | 29 | 12 | 27 s | 56 | 0%* | 25%* |
|  | IQR | Q1 | 11 | 72 | 1.13 | 1.93 | 24 | 9 | 24 s | 55 | 0% | 13% |
|  |  | Q3 | 37 | 101 | 1.51 | 3.51 | 30 | 17 | 41 s | 56 | 12% | 58% |

****Supplemental Figure 1. Histograms presenting score distributions of all balance measures included in the SiMPly Rehab testing battery. Balance scores of individuals presenting with CN III, IV and/or VI palsies are shown in red (n = 10). Scores achieved by the full sample are plotted in gray. One extreme outlier (> 3*IQR from Q1 or Q3) was excluded on the NIH-SBT_ratio1_ and TGT. Two extreme outliers were excluded on the WWTT-simple and WWTT-complex. NC: Assessment not completed.

Supplemental Figure 2. Histograms presenting score distributions of all balance measures included in the SiMPly Rehab testing battery. Balance scores of individuals presenting with CN VIII palsies are shown in orange (n = 3). Scores achieved by the full sample are plotted in gray. One extreme outlier (> 3*IQR from Q1 or Q3) was excluded on the NIH-SBT_ratio1_ and TGT. Two extreme outliers were excluded on the WWTT-simple and WWTT-complex. NC: Assessment not completed.

Supplemental Figure 3. Histograms presenting score distributions of all balance measures included in the SiMPly Rehab testing battery. Balance scores of individuals presenting with cerebellar signs (dysmetria, dysdiadochokinesia and/or ataxia) are shown in purple (n = 12).­­ Scores achieved by the full sample are plotted in gray. One extreme outlier (> 3*IQR from Q1 or Q3) was excluded on the NIH-SBT_ratio1_ and TGT. Two extreme outliers were excluded on the WWTT-simple and WWTT-complex. NC: Assessment not completed.

Supplemental Figure 4. Histograms presenting score distributions of all balance measures included in the SiMPly Rehab testing battery. Balance scores of individuals presenting with impaired proprioception are shown in green (n = 4). Scores achieved by the full sample are plotted in gray. One extreme outlier (> 3*IQR from Q1 or Q3) was excluded on the NIH-SBT_ratio1_ and TGT. Two extreme outliers were excluded on the WWTT-simple and WWTT-complex. NC: Assessment not completed.

Supplemental Figure 5. Histograms presenting score distributions of all balance measures included in the SiMPly Rehab testing battery. Balance scores of individuals at various times post-injury are shown. Participants were divided into: A) 2 weeks-1-month post-injury (n = 4, dark blue); B) >1 month to 3 months post-injury (n = 15, medium blue); and C) >3 months to 6 months post-injury (n = 18, light blue). One extreme outlier (> 3*IQR from Q1 or Q3) was excluded on the NIH-SBT_ratio1_ and TGT. Two extreme outliers were excluded on the WWTT-simple and WWTT-complex. NC: Assessment not completed.
